# Lung SBRT: dosimetric and delivery comparison of RapidArc, TomoTherapy, and IMRT

**DOI:** 10.1120/jacmp.v14i4.4065

**Published:** 2013-07-08

**Authors:** Ashleigh Weyh, Andre Konski, Adrian Nalichowski, Jordan Maier, Danielle Lack

**Affiliations:** ^1^ Karmanos Cancer Center Detroit MI; ^2^ Department of Radiation Oncology Wayne State University Detroit MI USA

**Keywords:** stereotactic body radiotherapy, RapidArc, helical tomotherpy, lung cancer

## Abstract

This study seeks to compare fixed‐field intensity‐modulated radiation therapy (FF IMRT), RapidArc (RA), and helical tomotherapy (HT) to discover the optimal treatment modality to deliver SBRT to the peripheral lung. Eight patients with peripheral primary lung cancer were reviewed. Plans were prescribed a dose of 48 Gy and optimized similarly with heterogeneity corrections. Plan quality was assessed using conformality index (${\text{CI}}_{100\%}$), homogeneity index (HI), the ratio of the 50% isodose volume to PTV ($R_{50\%}$) to assess intermediate dose spillage, and normal tissue constraints. Delivery efficiency was evaluated using treatment time and MUs. Dosimetric accuracy was assessed using gamma index (3% dose difference, 3 mm DTA, 10% threshold), and measured with a PTW ARRAY seven29 and OCTAVIUS phantom. ${\text{CI}}_{100\%},\text{HI}$, and $R_{50\%}$ were lowest for HT compared to seven‐field coplanar IMRT and two‐arc coplanar RA (p<0.05). Normal tissue constraints were met for all modalities, except maximum rib dose due to close proximity to the PTV. RA reduced delivery time by 60% compared to HT, and 40% when compared to FF IMRT. RA also reduced the mean MUs by 77% when compared to HT, and by 22% compared to FF IMRT. All modalities can be delivered accurately, with mean QA pass rates over 97%. For peripheral lung SBRT treatments, HT performed better dosimetrically, reducing maximum rib dose, as well as improving dose conformity and uniformity. RA and FF IMRT plan quality was equivalent to HT for patients with minimal or no overlap of the PTV with the chest wall, but was reduced for patients with a larger overlap. RA and IMRT were equivalent, but the reduced treatment times of RA make it a more efficient modality.

PACS numbers: 87.53.Ly87.55.N‐, 87.55.D‐, 87.56.bd

## I. INTRODUCTION

Lung cancer is the most common noncutaneous malignancy world‐wide and accounts for the most cancer deaths in both men and women.^(1)^ The current standard of care for early stage (I/II) lung cancer is surgical resection, which provides patients with a five‐year survival of 50%–70%.^(2)^ However, many patients are judged inoperable due to decreased cardiopulmonary reserve, cardiac dysfunction, diabetes mellitus, vascular disease, general frailty, and other comorbidities.

Stereotactic body radiation therapy (SBRT) is a curative treatment option for inoperable patients. Compared to the standard fractionation schedules used with conventional 3D conformal radiation therapy (3D CRT), SBRT has been shown to improve overall survival rates with excellent local control for stage I/II non‐small cell lung cancer (NSCLC).^(3)^ The increased use of SBRT has created the need to evaluate the treatment modalities available for hypofractionated delivery. The Radiation Therapy Oncology Group (RTOG) Protocol 0236 was a phase II trial investigating SBRT for patients with medically inoperable stage I/II peripheral NSCLC. This protocol established a common way for clinics to simulate, plan, and treat lung cancer with SBRT.^(4)^ Use of heterogeneity corrections (HC) in dose calculation and planning with intensity‐modulated radiation therapy (IMRT) were not allowed in RTOG 0236. However, at present, use of both is widespread in many clinics. HC are important for SBRT of the peripheral lung because there is much tissue inhomogeneity in the thorax, especially at the lung and chest wall interface where these peripheral lung tumors tend to develop. This inhomogeneity causes a decrease in plan quality which needs to be examined.^(5)^ In addition, there has been an increase in use of IMRT to treat SBRT of the lung,^3^, ^6^, ^7^, ^8^, ^9^ yet these different types of IMRT have not been fully evaluated. There are multiple ways to deliver IMRT SBRT treatments to the peripheral lung including fixed‐field IMRT (FF IMRT), helical tomotherapy (HT), and volumetric‐modulated arc therapy (VMAT). Varian RapidArc (RA) is one version of VMAT, and was used in this study. RA is an arc‐based IMRT technique that modulates the intensity of the beam as it rotates around the patient. RA has been reported to offer better organ sparing than FF IMRT for prostate and intracranial lesions.^10^, ^11^ HT has been used to treat cancer since the early 2000s, and also uses a full 360° treatment arc. Studies have shown HT can spare critical organs while improving target coverage, when compared to FF IMRT.^12^, ^13^, ^14^ Further investigations into the use of IMRT and HC for SBRT need to be done, and this study compares the ability of three modalities to utilize these technologies for SBRT delivery to the peripheral lung.

Currently our institution uses HT to deliver SBRT to the peripheral lung. Treatment times can be quite long, and have exceeded 20 minutes.^(15)^ Using multiple noncoplanar beams for 3D conformal SBRT or coplanar FF IMRT also results in treatment times exceeding 10 minutes.^(9)^ Lung SBRT treatments can be very uncomfortable for the patients due to the abdominal compression used for immobilization and patients’ poor pulmonary function. Therefore, an ideal modality would be able to deliver fast and accurate treatments without sacrificing plan quality. RA is known for its fast treatment times.^11^, ^16^, ^17^ There are few data demonstrating how well RA can deliver SBRT to a peripheral lung cancer.^18^, ^19^ The goal of this study is to compare RA, HT, and FF IMRT in the treatment of SBRT lung, with the inclusion of heterogeneity corrections. Plan quality, delivery efficiency, and dosimetric accuracy were assessed.

## II. MATERIALS AND METHODS

### A. Patient setup and simulation

Eight peripherally located early stage (T1/T2) NSCLC patients, previously treated with HT were selected for this study. Patients were simulated supine and immobilized by either a stereotactic body frame (CIVCO Medical Solutions, Orange County, CA) or a custom made alpha cradle (KGF Enterprise, Inc., Chesterfield, MI). At simulation, two image sets were taken; a free breathing CT and a 4D CT acquired using a pressure sensor belt (AZ‐773V; Anzai Medical Corp., Aichi, Japan) interfaced to a Siemens Sensation Open CT scanner (Siemens Corporation, Erlangen, Germany). The 4D CT dataset was used to generate the internal target volume, ITV. Planning and dose calculation were conducted on the free‐breathing CT.

### B. Planning criteria

For treatment planning, a 5 mm expansion was given to the ITV, to generate a planning target volume, PTV. The prescription dose was 48 Gy in 4 fractions and normalized to deliver the prescription dose to 95% of the PTV. Organs at risk (OARs) included the spinal cord, esophagus, heart, trachea, left and right lungs, as well as the ribs located within 5 cm of the PTV. Dose constraints used for OARs are presented in Table 1.^(2)^

**Table 1:** Dose constraints for organs at risk, OARs. $V_{20}$ represents the percent volume of lung tissue that receives 20 Gy

*OAR*	*Volume*	*Maximum Total Dose*
Spinal Cord	Any Point	26 Gy
Esophagus	Any Point	30 Gy
Heart	Any Point	34 Gy
Trachea	Any Point	34.8 Gy
Rib	Any Point	40 Gy
Lung	$V_{20}$ <10%

### C. Treatment planning

For each SBRT patient, plans were evaluated for each of the three treatment modalities: FF IMRT, RA, and HT. Since each patient had an original HT SBRT plan used for treatment, only initial FF IMRT and RA plans needed to be created retrospectively. Both the FF IMRT and RA plans were created using the Eclipse v.8.6 treatment planning system (Varian Medical Systems, Inc., Palo Alto, CA), while HT plans were created using the Hi·Art 3 treatment planning platform (TomoTherapy Inc., Madison, WI). Plans for all modalities used the same free breathing CT image set, and all structure sets were contoured within Eclipse.

In plan optimization, the Eclipse and Hi·Art 3 treatment planning systems use different optimization methods, as well as different cost functions. This means that planning constraints cannot be set exactly the same for the two planning systems. SBRT plans were originally created for tomotherapy delivery, using the Hi·Art 3 optimization platform. This platform limits the planner in the number of constraints that can be placed for a given structure simultaneously. Therefore, to optimize the plans as equally as possible, the initial constraints for the FF IMRT and RA plans were kept similar to those used for the original HT plan. Similar to another plan comparison study, after the initial comparison between modalities was complete, each plan was allowed a second phase of plan optimization in an effort to create the best plan, but following the second phase, no further changes were allowed.^(20)^

Both RA and FF IMRT plans were created for delivery on a Varian Clinac iX (Varian Medical Systems Inc.) treatment machine. This machine has a 120 leaf millennium MLC and a MLC leaf width of 5 mm at isocenter. FF IMRT plans consisted of seven coplanar six MV photon beams with a fixed dose rate of 400 MU/min. Beam gantry angles were placed to cover a 240° span around the PTV with 40° spacing. Optimization was run in “beamlet mode” for approximately 350 iterations, at which point the cost function had converged. RA plans consisted of two 360° rotation arcs, with the first field rotating clockwise from a gantry angle of 180.1° to 179.9° and the second field rotating counterclockwise. Collimator angles of 45° and 315° were used, respectively.

Achieving a high degree of target conformity while sparing nearby organs at risk can be very challenging, making the planning of SBRT lung cases quite complex. Studies have been reported for various treatment sites comparing single‐ and double‐arc plans, and have shown inferior results for single‐rc plans, especially in complex planning scenarios.^20^, ^21^ Thus, single‐arc plans were not evaluated in this study. For all RapidArc plans, a photon energy of 6 MV was used and a maximum dose rate of 600 MU/min was set. Plans were optimized within the “Arc Optimization” module of Eclipse, using the same initial dose‐volume histogram (DVH) constraints used in IMRT optimization. Plan optimization was run through all five MR levels allowing for convergence of the objective function for all plans. Following optimization of both RA and FF IMRT plans, final dose calculation was performed using the analytic anisotropic algorithm (AAA, Eclipse v. 8.6.15), and heterogeneity correction applied.

HT plans were generated using the tomotherapy treatment planning station version v.3.1.4.7, using 6 MV photons delivered at a nominal dose rate of 865 cGy/min at isocenter. For beam modulation, a 64‐leaf binary MLC is used with a leaf width of 6.25 mm projected at isocenter.

In helical delivery, there are three main parameters used in planning. These are the longitudinal field size equal to the axial thickness of the fan beam, pitch equal to the distance the couch travels per gantry rotation relative to the field size at the axis of rotation, and the modulation factor equal to the maximum leaf opening time relative to the average leaf opening time. For all plans used in this study, these were set to values of 2.5 cm, 0.143, and 2.0, respectively. Once initial parameters are set for each plan, a full beamlet dose calculation was run followed by 350 optimization iterations allowing for full convergence of the cost function. For final dose calculation, HT's convolution superposition dose calculation algorithm, which includes heterogeneity correction, was used.

### D. Plan quality evaluation

For all treatment plans, plan quality was evaluated by reviewing each dose distribution and calculating select dosimetric indices for the PTV and OARs from each dose‐volume histogram (DVH). For the PTV, plan conformality was assessed using three indices. Conformality of the prescription dose to the target volume was assessed using the ICRU conformity index, ${\text{CI}}_{\text{RI}}$.^(22)^ This index is defined as:

(1) $$CI_{RI}=\frac{V_{RI}}{TV}$$

where $V_{\text{RI}}$ represents the volume of tissue covered by the reference isodose (RI=100%), and *TV* represents the PTV. CI values closest to 1.0 indicate better conformity of dose to the target. Intermediate dose spillage and falloff gradient beyond the PTV was assessed using two indices: $R_{50\%}$ and $D_{2\text{cm}}$. The $R_{50\%}$ index is also calculated according to Eq. (1), with a reference isodose equal to 50% the prescription dose. $D_{2\text{cm}}$ represents the maximum cumulative dose (as a percentage of the prescription dose) to any point located 2 cm away from the PTV. Lower $R_{50\%}$ ratios and lower $D_{2\text{cm}}$ doses indicate greater dose falloff and better plan conformity. Additionally, the global maximum dose for each plan was tracked. With respect to OARs, the maximum dose index to the spinal cord, esophagus, heart, trachea, and rib was tracked; while for lung, the $V_{20}$, or percent volume that receives 20 Gy, was recorded. Homogeneity index (HI), was also calculated for plan comparison. Homogeneity index is defined as:

(2) $$HI_{5/95}=\frac{D_{5\%}}{D_{95\%}}$$

where $D_{5\%}$ and $D_{95\%}$ are the minimum doses delivered to 5% and 95% of the PTV, respectively.^(23)^ Values of HI closest to 1 indicate greater homogeneity within the target.

### E. Delivery efficiency

Delivery efficiency was evaluated using total plan MUs and delivery time. Delivery times were defined from beam‐on of the first field to beam‐off of the last field. The delivery times included programming subsequent beams, as well as collimator and gantry rotations. For RA and FF IMRT treatments, plans were delivered in quality assurance (QA) mode using the MOSAIQ record and verify (R&V) system (IMPAC Medical Systems, Sunnyvale, CA). Beams were delivered as planned, allowing for the delivery time to be measured directly. Since HT treatments are time‐based deliveries, treatment times were taken directly from the plan report.

### F. Dosimetric accuracy

Dosimetric accuracy was assessed by measuring the 2D dose distribution of the patient plan in a solid water phantom. The OCTAVIUS phantom and ARRAY seven29 ion chamber (PTW, Freiburg, Germany) were used for all measurements. The measured cumulative 2D dose plane was compared with the calculated dose distribution calculated on the OCTAVIUS phantom by both the Eclipse and tomotherapy treatment planning systems. Data were analyzed with VeriSoft v.4.0 (PTW) software to determine the gamma pass rate (criteria: 3% max dose difference, 3 mm distance to agreement, 10% threshold). Seven29 was cross‐calibrated by delivering 2 Gy to the center chamber with a set number of MUs (or time in the HT case).

## III. RESULTS

**Figure 1:**
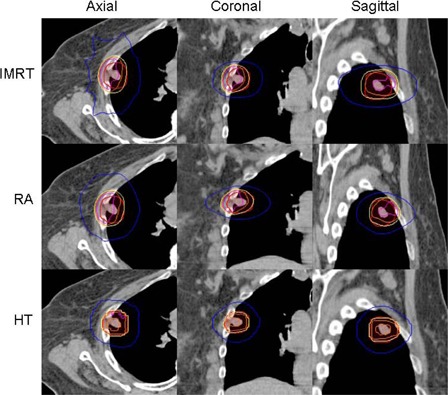
From top to bottom: FF IMRT, RA, and HT. Axial, coronal, and sagittal views from one representative patient. Projected isodose lines are 110% (pink), 105% (orange), 100% (yellow), and 50% (blue) isodose lines.

Dosimetric comparison results are shown in Table 2, and treatment efficiency results are shown in Table 3. A paired *t*‐test was used to compare the mean results of each modality. A p‐value <0.05 was the threshold for significance. Axial, coronal, and sagittal isodose distributions from FF IMRT, RA, and HT plans are shown for one representative patient in Fig. 1.

**Table 2:** Summary of dosimetric comparison expressed in mean values ± standard deviation. Significance is indicated by p<0.05. Only $D_{2\text{cm}}$ and Rib $D_{\text{max}}$ were significant for RA vs. FF IMRT

	*IMRT*	*RA*	*HT*	*P‐value RA vs. HT*	*P‐value FF IMRT vs. HT*
Target (PTV)					
${\text{CI}}_{100\%}$	1.36±2.9	1.29±2.2	1.06±0.03	<0.05	<0.05
$R_{50\%}$	7.1±1.9	6.53±1.2	5.3±0.9	<0.01	<0.01
$D_{2\text{cm}}(\%)$	75.65±12.7	67.34±10.5	51.89±16.3	<0.01	<0.01
HI	1.15±0.02	1.14±0.02	1.07±0.02	<0.01	<0.01
Maximum Dose (%)	118.26±2.3	117.51±1.0	110.26±2.9	<0.01	<0.01
OAR (Gy)					
Spinal Cord	13.9±9.7	14.25±7.7	13.08±5.6	0.22	0.22
Esophagus	11.62±5.7	14.12±6.5	13.71±6.3	0.54	0.51
Heart	10.93±12.2	9.72±11.9	8.59±10.3	0.12	0.06
Trachea	11.32±8.3	11.86±8.3	11.66±6.9	0.81	0.77
Rib $D_{\text{max}}$	49.53±10.5	47.04±12.9	42.54±12.1	<0.01	<0.01
Lung $V_{20}(\%)$	6.6±3.7	6.32±3.9	5.81±3.4	0.11	0.12

**Table 3:** Delivery efficiency summarized by patient

	*Total MUs*			*Delivery Time (sec)*			*QA Pass Rate*		
*Patient Number*	*RA*	*HT*	*IMRT*	*RA*	*HT*	*IMRT*	*RA*	*HT*	*IMRT*
1	3493	15460	4352	388	1092	778	100.0%	92.9%	99.4%
2	3856	12080	3359	440	862	680	100.0%	97.9%	100.0%
3	3707	18684	3040	414	1313	574	98.7%	98.9%	97.3%
4	2993	10273	2201	356	738	462	100.0%	97.6%	100.0%
5	2903	18483	3087	348	1299	591	100.0%	93.6%	100.0%
6	3027	13261	2640	359	942	512	100.0%	98.0%	99.4%
7	1855	9316	5262	394	647.1	939	100.0%	100.0%	98.2%
8	3643	10542	4990	423	730.8	781	98.3%	100.0%	99.2%
Mean	3184.6	13512.4	3616.4	390.3	953.0	664.6	99.6%	97.4%	99.2%
Std. Dev.	647.8	3670.1	1119.4	33.9	258.1	160.3	0.7%	2.7%	1.0%
RA vs. HT		p≤0.01			p≤0.01			p=0.08	
HT vs. FF IMRT		p≤0.01			p=0.06			p=0.16	
RA vs. FF IMRT		p=0.65			p≤0.01			p=0.19	

### A. Plan evaluation

**Figure 2:**
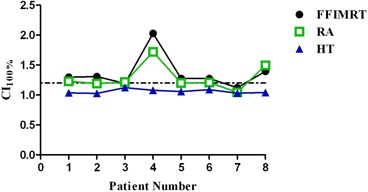
Conformity index (${\text{CI}}_{100\%}$) results for all patients. HT was lowest for all patients in study. Change in graph trend for patients 4 and 8 possibly explained by great overlap of patient's PTV with the chest wall. Dotted line represents ${\text{CI}}_{100\%}=1.2$.

**Figure 3:**
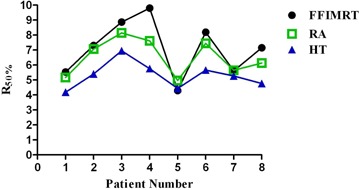
$R_{50\%}$ results for all eight patients. On average, HT was significantly lower in six out of eight patients. Changes in graph trend for patients 5 and 7 could possibly be explained by location of these tumors away from the chest wall.

The average PTV for this patient group was 39.34 cc (range: 7.63–83.43) with a standard deviation of 27.75 cc. HT was statistically significant when compared to both two‐arc coplanar RA and seven‐field coplanar IMRT, for ${\text{CI}}_{100\%},R_{50\%},D_{2\text{cm}},\text{HI}$, and maximum dose (p<0.05). RA and FF IMRT were comparable, with results significant only for $D_{2\text{cm}}$, when comparing RA and FF IMRT (p<0.05). HT produced the most conformal plans, demonstrated by the $R_{50\%}$ and $D_{2\text{cm}}$. Mean ${\text{CI}}_{100\%}$ for HT was closest to unity (1.06); values for RA and FF IMRT were 21% and 28% higher, respectively. Results for ${\text{CI}}_{100\%}$ are plotted in Fig. 2. Mean $R_{50\%}$ was lowest for HT with a value of 5.30. $R_{50\%}$ values for RA and FF IMRT were on average 23% and 34% higher, respectively. Results for $R_{50\%}$ can be seen in Fig. 3. At a distance of 2 cm from the PTV, HT plans produced the lowest doses, indicating the least amount of intermediate dose spillage. On average, the $D_{2\text{cm}}$ for HT plans was 51.89% of the prescription dose. For RA and FF IMRT plans, the $D_{2\text{cm}}$ was 67.34% and 75.25%, respectively. HT created the most uniform dose distribution in the PTV(HI=1.07), followed by RA (1.14) and FF IMRT (1.15). Mean maximum doses for HT were lowest (110.26%), as compared to FF IMRT (118.26%) and RA (117.51%).

**Figure 4:**
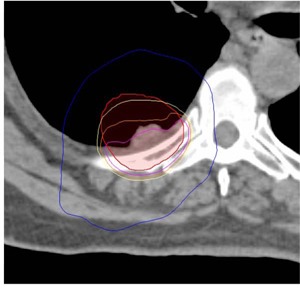
Isodose distribution in PTV at rib/chest wall interface. Isodose line projected: 110% (pink) 105% (orange) 100% (yellow) and 50% (blue) 110% hotspot falls almost entirely in rib/chest wall.

Critical structure doses for all three modalities are summarized in Table 2. All modalities met most of the normal tissue constraints and with similar mean values which were not statistically significant (p>0.05). Only the dose tolerance to the rib was exceeded. In four cases, the PTV either encompassed part of the rib or was in close proximity (<1mm) to the rib, such that the rib received 48 Gy or higher. Being the most conformal, HT plans provided the lowest doses (42.54 Gy) to the rib (p<0.01), followed by RA (47.04 Gy) and FF IMRT (49.53 Gy). This can be seen upon visual examination of isodose lines in Figs. 1 and 4.

### B. Delivery efficiency

RA provided the most efficient delivery, with a mean time of 390.3 seconds (p<0.01), compared to FF IMRT and HT. FF IMRT was the second fastest and had a mean time of 664.6 seconds, and HT had the longest mean time of 934.2 seconds.

### C. Dosimetric accuracy

Gamma pass rates for all modalities were >97% demonstrating that each modality could be delivered accurately. RA had the highest mean QA pass rate of 99.6%, followed by FF IMRT with a mean pass rate of 99.2% and by HT with a mean of pass rate of 97.4%. All the measurements were in line with our clinical patient QA data. A significant difference did not exist between modalities.

## IV. DISCUSSION

In this study, all three modalities were able to deliver highly conformal SBRT plans. Due to the location of peripheral lung tumors, most OARs are removed from the area of high‐dose gradient and the dose constraints are easily met. However, when choosing the superior modality, clinics need to analyze plans specifically for each patient to determine which plan will give the best results. The superior modality for each dosimetric criterion can vary based on patient anatomy, tumor location, and tumor size. No one modality was able to produce the best result for every measure of plan quality across one single patient.

Our study found that HT plans achieved the best dose conformity of the PTV, as demonstrated by the conformality indices reviewed (${\text{CI}}_{100\%},R_{50\%},D_{2\text{cm}}$) where values were lowest for HT. Additionally, HT plans were the most uniform in the PTV, with HI indices closest to unity. Results were significant between HT and seven‐field coplanar IMRT, and between HT and two‐arc coplanar RA for all categories of target conformality (p<0.05). Despite HT being the most conformal, all three modalities struggled with intermediate dose spillage, which was measured by $R_{50\%}$ and $D_{2\text{cm}}$. The mean $D_{2\text{cm}}$ value for FF IMRT was 50% higher than HT and 30% higher than RA. A low $R_{50\%}$ was difficult to achieve with all three modalities.

The uniformity of the HT plans is also evident by visual evaluation of isodose distributions, as shown in Fig. 1. The HT planning system was able to create the most homogeneous and uniform distribution throughout the tumor, even in areas of great tissue inhomogeneity. The majority of plan hot spots were located within the PTV, which is seen as an advantage by TG‐101.^(24)^ The FF IMRT and RA plans were more likely to have hot spots located along the periphery of the PTV or partially outside of the PTV, lowering plan quality. The maximum dose seen in the IMRT and RA plans were on average 7%–8% higher than HT. When evaluating the isodose distributions of the Eclipse FF IMRT and RA plans, there was a visible disproportion in the distributions. At the interface of the rib and the lung, where there is a large difference in tissue densities, large hot spots were observed in the rib. Conversely, cold spots on the tumor were observed at the lung/tumor interface. Figure 4 demonstrates this effect in one representative patient, where the PTV has much overlap with the rib and chest wall. This effect occurred in RA and FF IMRT planned patients who had overlap of the PTV and rib/chest wall.

Four of the eight patients had overlap of the PTV with the chest wall, and a trend was observed when comparing plans of patients with and without PTV overlap. Figure 2 plots results for ${\text{CI}}_{100\%}$, and patients 4 and 8 both have increases in ${\text{CI}}_{100\%}$ for FF IMRT and RA plans. These patients’ PTV had a much greater overlap with the chest wall than the other patients in this study, with 44% and 40% of their PTV consisting of soft tissue and the chest wall, respectively. Another trend can be observed in Fig. 3, which plots $R_{50\%}$ results. Only patient 5 produced a FF IMRT plan with a $R_{50\%}$ similar to HT, while all other patients’ HT plans achieved a much lower $R_{50\%}$ when compared to RA and FF IMRT. Patient 5's PTV had only a 15.5% overlap with the chest wall, resulting in a more homogenous PTV. Patient 7 also had less PTV overlap (35%), and the $R_{50\%}$ values of the FF IMRT, RA, and HT plans were much closer in value than those of the other six patients. Based on this result, it is observed that FF IMRT and RA can achieve a comparable $R_{50\%}$ to HT when the PTV is more homogenous. These trends show that the quality of FF IMRT and RA plans created in the Varian Eclipse treatment planning system are much more dependent on heterogeneity of the tissues surrounding the PTV than the HT plans.

The reason why plans generated with the Eclipse treatment planning system are more dependent on tissue inhomogeneities stems from differences in the dose calculation algorithms used for optimization and final dose calculation. For optimization, a multiresolution dose calculation (MRDC) algorithm is used. This algorithm is simpler and faster than the anisotropic analytical algorithm (AAA) used for final dose calculation. However, the increased speed gained by the MRDC algorithm comes with a decreased ability to predict dose in heterogeneous tissue.^(25)^ The decreased accuracy of the MRDC algorithm for low‐density tissues makes FF IMRT and RA planning for SBRT more difficult, and results in the FF IMRT and RA plans generated in this study being less uniform and conformal, as compared to the HT plans.

There are many critical organs located in the thorax, and sparing of normal tissues is of great interest. Due to the somewhat isolated location of most peripheral lung tumors, most patients’ OARs were located outside of the high‐dose region. The rib was the only OAR for which all modalities could not meet the planning criteria in every instance. Large doses to the rib need to be further evaluated, based on recent evidence of rib fractures reported after SBRT treatments to the chest.^(26)^ In our study, cases where the PTV overlapped or abutted the rib had a maximum rib dose of 48 Gy or higher. This was further complicated in the Eclipse planned RA and FF IMRT treatments, where many of the hot spots fell in the rib portion of the PTV. Rib doses need to be minimized without sacrificing coverage of the PTV, and it has been shown that a steep dose gradient outside the PTV can reduce the risk of rib fracture.^(27)^ When compared to seven‐field IMRT and two‐arc RA, HT gives the best chance for sparing of the rib because the dose distributions created were the most conformal and uniform.

In this study, RA treatment times were reduced by approximately 40% compared to FF IMRT. Treatment times were reduced by approximately 60% when comparing RA to HT. For HT, treatment times could be reduced by using larger pitch and/or lowering the modulation factor. However, by changing these parameters, the plan quality may be compromised. Plan MUs for RA were reduced by 22% when compared to FF IMRT, and reduced by 77% when compared to HT. Intrafraction motion is a very crucial concern when delivering an SBRT plan. In order to deliver the plan as accurately as possible, respiratory motion and patient movement due to poor pulmonary function must both be accounted for and or minimized. Image guidance is required before SBRT treatments, further increasing already long treatment times. Reducing treatment times for SBRT delivery could be very beneficial to patients. Shorter treatment times will improve patient comfort and reduce patient motion during treatment, providing an overall more accurate treatment.

Other modality comparison studies have been conducted, but none so far have directly compared all three modalities, FF IMRT, RA, and HT, for SBRT of the peripheral lung. Since SBRT lung treatments are difficult to plan, a more comprehensive look at this treatment needed to occur. The general finding of the other comparison studies, for multiple treatment sites, have shown that RA is equivalent to FF IMRT and HT in terms of PTV conformality and critical organ sparing. When treatment time is also considered, RA has sometimes been seen as the superior modality.^10^, ^23^ However, the results of this study did not fully agree with these previous findings. Inclusion of heterogeneity corrections, especially in anatomic locations with large differences in tissue homogeneity, can negatively affect plan quality of Eclipse RA and IMRT plans; therefore comparisons need to correct for heterogeneities to be fair. Location of the tumor, volume of the PTV, surrounding organs at risk, and other anatomical considerations of the patient all factor into generating the best treatment plan.

The limitations of the study include potential systematic errors due to differences among optimization algorithms and statistics due to low patient number evaluated in this study.

Additionally, the comparison results drawn from this study are specific to the three ways of planning — FF IMRT, RA, and HT — for SBRT lung treatments evaluated. For FF IMRT, a seven‐field coplanar arrangement was used and for RA a two‐arc coplanar beam configuration was examined. Alternatively, these modalities could have been planned using more beams or a noncoplanar beam arrangement. Similarly, for HT, a field size of 1.0 cm could have been used instead of a 2.5 cm field size. Incorporating these changes would increase plan complexity and that could improve the dosimetric quality of each modality at the expense of treatment time. Long‐term patient outcomes and results, along with further studies similar to this one, are needed before definite conclusions about the comparison of modalities can be made.

## V. CONCLUSIONS

For lung SBRT treatments, HT performed better dosimetrically as compared to seven‐field coplanar IMRT and two‐arc coplanar RA, reducing maximum rib dose, as well as improving dose conformity and uniformity. RA and FF IMRT plan quality was equivalent to HT for patients with minimal or no overlap of the PTV with the chest wall, but was reduced for patients with a larger overlap. RA and IMRT were equivalent, but the reduced treatment times of RA make it a more efficient modality.

## ACKNOWLEDGMENTS

The authors would like to thank Dr. Michael Snyder for his help editing the manuscript.
